# Barium chloride injures myofibers through calcium-induced proteolysis with fragmentation of motor nerves and microvessels

**DOI:** 10.1186/s13395-019-0213-2

**Published:** 2019-11-06

**Authors:** Aaron B. Morton, Charles E. Norton, Nicole L. Jacobsen, Charmain A. Fernando, D. D. W. Cornelison, Steven S. Segal

**Affiliations:** 1 Department of Medical Pharmacology and Physiology, University of Missouri, MA415 Medical Sciences Building, 1 Hospital Drive, Columbia, MO 65212 USA; 2 Division of Biological Sciences and Christopher S. Bond Life Sciences Center, University of Missouri, Columbia, MO 65201 USA; 3 Dalton Cardiovascular Research Center, Columbia, MO 65211 USA

**Keywords:** Skeletal muscle, Motor innervation, Capillary supply, Neuromuscular junction

## Abstract

**Background:**

Local injection of BaCl_2_ is an established model of acute injury to study the regeneration of skeletal muscle. However, the mechanism by which BaCl_2_ causes muscle injury is unresolved. Because Ba^2+^ inhibits K^+^ channels, we hypothesized that BaCl_2_ induces myofiber depolarization leading to Ca^2+^ overload, proteolysis, and membrane disruption. While BaCl_2_ spares resident satellite cells, its effect on other tissue components integral to contractile function has not been defined. We therefore asked whether motor nerves and microvessels, which control and supply myofibers, are injured by BaCl_2_ treatment.

**Methods:**

The intact extensor digitorum longus (EDL) muscle was isolated from male mice (aged 3–4 months) and irrigated with physiological salt solution (PSS) at 37 °C. Myofiber membrane potential (V_m_) was recorded using sharp microelectrodes while intracellular calcium concentration ([Ca^2+^]_i_) was evaluated with Fura 2 dye. Isometric force production of EDL was measured in situ, proteolytic activity was quantified by calpain degradation of αII-spectrin, and membrane disruption was marked by nuclear staining with propidium iodide (PI). To test for effects on motor nerves and microvessels, tibialis anterior or gluteus maximus muscles were injected with 1.2% BaCl_2_ (50–75 μL) in vivo followed by immunostaining to evaluate the integrity of respective tissue elements post injury. Data were analyzed using Students *t* test and analysis of variance with *P* ≤ 0.05 considered statistically significant.

**Results:**

Addition of 1.2% BaCl_2_ to PSS depolarized myofibers from − 79 ± 3 mV to − 17 ± 7 mV with a corresponding rise in [Ca^2+^]_i_; isometric force transiently increased from 7.4 ± 0.1 g to 11.1 ± 0.4 g. Following 1 h of BaCl_2_ exposure, 92 ± 3% of myonuclei stained with PI (vs. 8 ± 3% in controls) with enhanced cleavage of αII-spectrin. Eliminating Ca^2+^ from PSS prevented the rise in [Ca^2+^]_i_ and ameliorated myonuclear staining with PI during BaCl_2_ exposure. Motor axons and capillary networks appeared fragmented within 24 h following injection of 1.2% BaCl_2_ and morphological integrity deteriorated through 72 h.

**Conclusions:**

BaCl_2_ injures myofibers through depolarization of the sarcolemma, causing Ca^2+^ overload with transient contraction, leading to proteolysis and membrane rupture. Motor innervation and capillarity appear disrupted concomitant with myofiber damage, further compromising muscle integrity.

## Background

Acute injury to skeletal muscle initiates a coordinated process of tissue degeneration and regeneration that encompasses inflammation; digestion of damaged components; activation, proliferation, and differentiation of resident myogenic stem cells (satellite cells); and maturation of nascent myofibers [1, 2]. While different injury models (freeze injury, cardiotoxin, Marcaine™, and BaCl_2_) induce variable degrees of tissue damage and inflammation [3–5], the advantages of chemical injury with BaCl_2_ include both ease of use and its ability to reproducibly damage myofibers while preserving their associated satellite cells [3, 6–9] (Additional file 1). However, the mechanism by which BaCl_2_ exposure leads to the death of skeletal muscle myofibers has not been identified. The divalent cation Ba^2+^ blocks inward rectifying potassium channels (K_IR_) at concentrations of 10–100 μM [10] and serves as a broad spectrum K^+^ channel inhibitor at concentrations ≥ 1 mM [11, 12]. Thus, injection of 1.2% BaCl_2_ (~ 57 mM), as used to induce muscle damage [3, 6–9], would be predicted to depolarize myofibers. In turn, depolarization can activate L-type voltage-gated calcium channels (Ca_V_1.1) in the sarcolemma, leading to increases in intracellular Ca^2+^ concentration ([Ca^2+^]_i_) via release from the sarcoplasmic reticulum and influx from the extracellular fluid [13, 14]. Sufficient elevation of [Ca^2+^]_i_ initiates proteolysis, leading to degradation of contractile proteins and cell membranes [15, 16]. We therefore tested the hypothesis that BaCl_2_ injures skeletal muscle through myofiber depolarization, with elevated [Ca^2+^]_i_ leading to proteolysis and rupture of the sarcolemma.

Motor axons and microvessels are intimately associated with myofibers. At the neuromuscular junction (NMJ), myelinated axons projecting from α motor neurons in the spinal cord terminate and are covered by perisynaptic Schwann cells which overlay postsynaptic clusters of nicotinic acetylcholine receptors [17]. Arterioles control the perfusion of capillary networks that collectively span the entire length of myofibers to provide oxygen and nutrients essential to supporting contractile activity [18]. While BaCl_2_ can damage the capillary supply [3], it is unknown whether concomitant injury occurs to the motor nerves that control myofiber contraction. Therefore, also we tested whether local injection of BaCl_2_ disrupts the integrity of NMJs and capillaries concomitant with injuring myofibers.

## Methods

### Aim, design, and setting

The aim of this study was to determine how BaCl_2_ injures skeletal muscle myofibers and whether motor innervation and microvascular supply are concomitantly disrupted by exposure to BaCl_2_. We studied the acute effects of BaCl_2_ exposure on membrane potential (V_m_), [Ca^2+^]_i_, membrane integrity, αII-spectrin degradation, and force production in extensor digitorum longus (EDL) muscles. Neuromuscular synapses were studied in the tibialis anterior (TA) muscle and the microcirculation was studied in the gluteus maximus (GM) muscle at 0 (control) and 1–3 days post injury (dpi) following local injection of BaCl_2_.

### Animal care and use

All protocols and experimental procedures were reviewed and approved by the Animal Care and Use Committee of the University of Missouri (Columbia, MO, USA). Procedures were performed on male mice (age, 3–4 months; weight, ~ 30 g) of the following strains: C57BL/6J (WT) (Jackson Labs, *n* = 31), D2-Tg(*S100B*-EGFP)1Wjt/J (*S100B*-GFP) (Jackson Labs, *n* = 6); and *Cdh5-*CreER^T2^:*ROSA-*mTmG (*Cdh5*-mTmG) (cross of VE-cadherin-CreER^T2^ mice [19] and *Rosa26*^mTmG^ mice (007676 Jackson Labs)), (*n* = 3). Cre recombination was induced through intraperitoneal injection of 100 μg tamoxifen (Cat. # T5648, Sigma-Aldrich; St. Louis, MO, USA; 1 mg/100 μL in peanut oil) on 3 consecutive days with at least 1 week allowed prior to further study. Mice were housed locally on a 12-h light-dark cycle at ~ 23 °C, with freshwater and food available ad libitum.

To induce muscle injury in vivo, mice were anesthetized with ketamine and xylazine (100 mg/kg and 10 mg/kg, respectively; intraperitoneal injection), the skin was shaved over the muscle of interest, and then 1.2% BaCl_2_ was injected unilaterally into the TA (50 μL [9]) or under the GM (75 μL [8]) as described. Mice were kept warm during recovery and then returned to their cage. On the day of an experiment, mice were anesthetized (as above). Following tissue harvest, mice were killed by exsanguination.

### Membrane potential

The EDL muscle was used for these experiments because it can be isolated and secured in vitro by its tendons to approximate in vivo muscle length without damaging myofibers. An EDL muscle was removed from the anesthetized mouse, pinned onto transparent rubber (Sylgard 124; Midland, MI, USA), placed in a tissue chamber (RC-37 N; Warner Instruments; Hamden, CT, USA), transferred to the stage of a Nikon 600FN microscope (Tokyo, Japan), and irrigated (3 mL min^− 1^) with standard physiological salt solution (PSS; pH 7.4) of the following composition: 140 mM NaCl (Fisher Scientific; Pittsburgh, PA, USA), 5 mM KCl (Fisher), 1 mM MgCl_2_ (Sigma), 10 mM HEPES (Sigma), 10 mM glucose (Fisher), and 2 mM CaCl_2_ (Fisher) while maintained at 37 °C.

The membrane potential (V_m_) of myofibers was recorded with an amplifier (AxoClamp 2B, Molecular Devices; Sunnyvale, CA, USA) using sharp microelectrodes pulled (P-97, Sutter Instruments; Novato, CA, USA) from glass capillary tubes (GC100F-10, Warner, Hamden, CT, USA) filled with 2 M KCl (~ 150 MΩ) with a Ag/AgCl pellet serving as a reference electrode [20]. The amplifier was connected to a data acquisition system (Digidata 1322A, Molecular Devices) and an audible baseline monitor (ABM-3, World Precision Instruments; Sarasota, FL, USA). Successful impalements were indicated by sharp negative deflection of V_m_, stable V_m_ for > 1 min, and prompt return to ~ 0 mV upon withdrawal of the electrode. Data were acquired at 1 kHz on a personal computer using AxoScope 10.1 software (Molecular Devices). Once a single myofiber was impaled, V_m_ was recorded for at least 5 min to establish a stable baseline. PSS containing 1.2% BaCl_2_ then irrigated the muscle at 37 °C. Additional experiments were performed using isotonic substitution of BaCl_2_ for NaCl (final [NaCl] = 54 mM vs. 140 mM in standard PSS) to test whether differences in osmolality affected responses during exposure to 1.2% BaCl_2_. Each of these experiments represents one myofiber in one EDL; each muscle was obtained from a separate mouse.

### Calcium photometry

Intracellular [Ca^2+^] responses were measured as reported [20]. An EDL muscle was incubated in either standard PSS or in Ca^2+^-free PSS containing 3 mM (ethylene glycol-bis (β-aminoethyl ether)-N,N,N′,N′-tetraacetic acid (EGTA)). Each solution contained 1 μM Fura 2-AM (Cat. # F4185, Fisher); a muscle was incubated for 60 min at 37 °C and then washed for 20 min to remove excess dye. The 1.2% BaCl_2_ was then added while fluorescence was recorded at 510 nm during alternative excitation (10 Hz) at 340 nm and 380 nm using a × 20 objective (Nikon Fluor20, numerical aperture (NA) = 0.45). Data were acquired using IonWizard 6.3 software (IonOptix; Milford, MA) on a personal computer and expressed as fluorescence (F) ratios (F_340_/F_380_) after subtracting autofluorescence recorded prior to dye loading.

### Membrane damage

As an index of myofiber membrane damage, EDL muscles were treated for 1 h with standard PSS, 1.2% BaCl_2_ dissolved in standard PSS, or 1.2% BaCl_2_ dissolved in Ca^2+^-free PSS then stained for 20 min with membrane-permeant Hoechst 33342 (1 μM, Cat. # H1399, Fisher) and membrane-impermeant propidium iodide (PI; 2 μM, Cat. # P4170, Sigma) in PSS. These dyes stain the nuclei of all cells and nuclei of cells with disrupted membranes, respectively [21]. Muscles were then washed for 30 min in standard PSS and image stacks were acquired with a water immersion objective (× 40; NA = 0.8) coupled to a DS-Qi2 camera with Elements software (version 4.51) on an E800 microscope (all from Nikon). Stained nuclei were counted within a defined region of interest (ROI; 300 × 400 μm) of image stacks using Image J (NIH) to quantify the percentage (%) of total nuclei stained with PI.

### Western blot for αll-spectrin degradation

EDL muscles were secured to approximate in situ length and incubated in either standard PSS or 1.2% BaCl_2_ in PSS for 1 h at 37 °C then frozen in liquid nitrogen. Following homogenization, protein concentration of the supernatant was quantified with the Bradford method (Cat. # 5000006; Sigma). Protein concentration of each sample was normalized in 4x Laemmli sample buffer (Cat. # 1610747, Bio-Rad; Hercules, CA, USA) containing 5% dithiothreitol. Samples were loaded on 4–20% gradient Mini-Protein TGX gels (Bio-Rad) for electrophoresis and transferred to LF-PVDF membranes (Millipore; Burlington, MA). Following 2 h blocking in 5% milk, membranes were incubated overnight at 4 °C and again for 3 h at 25 °C in primary antibody raised against αII-spectrin (1:250, Cat. # sc48382, Santa Cruz; Dallas, TX, USA). A secondary antibody (Alexa Fluor 800 IgG, 1:5000; Cat. # 926–32,212, Li-Cor Biosciences; Lincoln, NE, USA) was used to quantify protein differences with an Li Cor Odyssey Fc imaging system. Western blots were normalized to total protein according to the recommendations for fluorescent Western blotting [22] using Revert total protein stain (Cat. # 926-11010, Li-Cor). The 40 kDa bands correlate with the total protein in each lane and are shown to represent equal protein loading [23, 24].

### Muscle force

The EDL was prepared for in situ measurements as described [25]. Briefly, in an anesthetized mouse, a 2-0 suture was placed around the left patellar tendon. The distal tendon of the EDL was isolated, secured in 2-0 suture, and then severed from its insertion. The mouse was placed prone on a plexiglass board and the patellar tendon was secured to a vertical metal peg immobilized in the board. The distal EDL tendon was tied to a load beam (LCL-113G; Omega, Stamford, CT, USA) coupled to a Transbridge amplifier (TBM-4; World Precision Instruments, Sarasota, FL, USA). The load beam was attached to a micrometer for adjusting optimal length (*L*_o_) as determined during twitch contractions at 1 Hz [8]. A strip of KimWipe® was wrapped around the EDL and 1.2% BaCl_2_ irrigated the EDL (3 mL min^− 1^) while resting force was evaluated for 1 h with Power Lab acquisition software (ADInstruments, Colorado Springs, CO, USA) on a personal computer.

### Neuromuscular junction histology

In a mouse strain with genetically labeled Schwann cells (*S100B*-GFP/Kosmos [26]), the TA muscle of one hindlimb was injured with BaCl_2_ injection and the contralateral limb was left intact. Mice were studied at 0 (control), 1, 2, and 3 dpi. At each time point, the hindlimb was excised, the TA was removed, and myofibers were gently teased apart with fine forceps in ice-cold phosphate-buffered saline (PBS, pH 7.4) to facilitate antibody penetration. Samples were fixed for 15 min in 4% paraformaldehyde, washed in PBS 3 times for 5 min, and stained for neurofilament-heavy (primary antibody: chicken anti-mouse, 1:400; Cat. # CPCA-NF-H Encor Biotechnology Inc.; Gainesville, FL, USA; secondary antibody: goat anti-chicken, 647 IgY, 1:1000, Cat. # A-21449, Fisher); each antibody was incubated overnight at 4 °C followed by washing in PBS 6 times for 30 min. Nicotinic receptors were then stained with α-bungarotoxin conjugated to tetramethylrhodamine (1:500, Cat. # 00014, Biotrend; Koln, Germany) for 2 h at room temperature and washed in PBS prior to imaging. Images were acquired with a × 25 water immersion objective (NA = 0.95) at × 1.75 digital zoom on an inverted laser scanning confocal microscope (TCS SP8, Leica Microsystems Buffalo Grove, IL, USA) using Leica LAX software. Image stacks (thickness, ~ 150 μm) were used to resolve NMJ morphology.

### Microvessel histology

The GM was used for histological analysis of skeletal muscle microvasculature based on it being a thin (100–200 μm), planar muscle which facilitates imaging of microvessels throughout the tissue [8]. A GM was dissected away from its origin along the lumbar fascia, sacrum, and iliac crest, reflected away from the body, and spread onto a transparent rubber pedestal. Superficial connective tissue was removed using microdissection and the muscle was severed from its insertion. To image capillary networks, the unfixed GM was immersed in PBS, a small glass block was placed on top to gently flatten the muscle, and image stacks were acquired as described for NMJs. In *Cdh5*-mTmG mice, all endothelial cells are labeled with membrane-localized GFP following tamoxifen-induced Cre recombination.

### Data analysis

Data were analyzed using Student’s *t* test and one-way Analysis of Variance with Bonferroni’s multiple comparison test post hoc when appropriate (Prism 5, GraphPad Software, La Jolla, CA, USA). Summary data are presented as means ± SEM; *n* refers to the number of preparations (each from a different mouse) in a given experimental group. *P* ≤ 0.05 was considered statistically significant.

## Results

### BaCl_2_ depolarizes myofibers

**Fig. 1:**
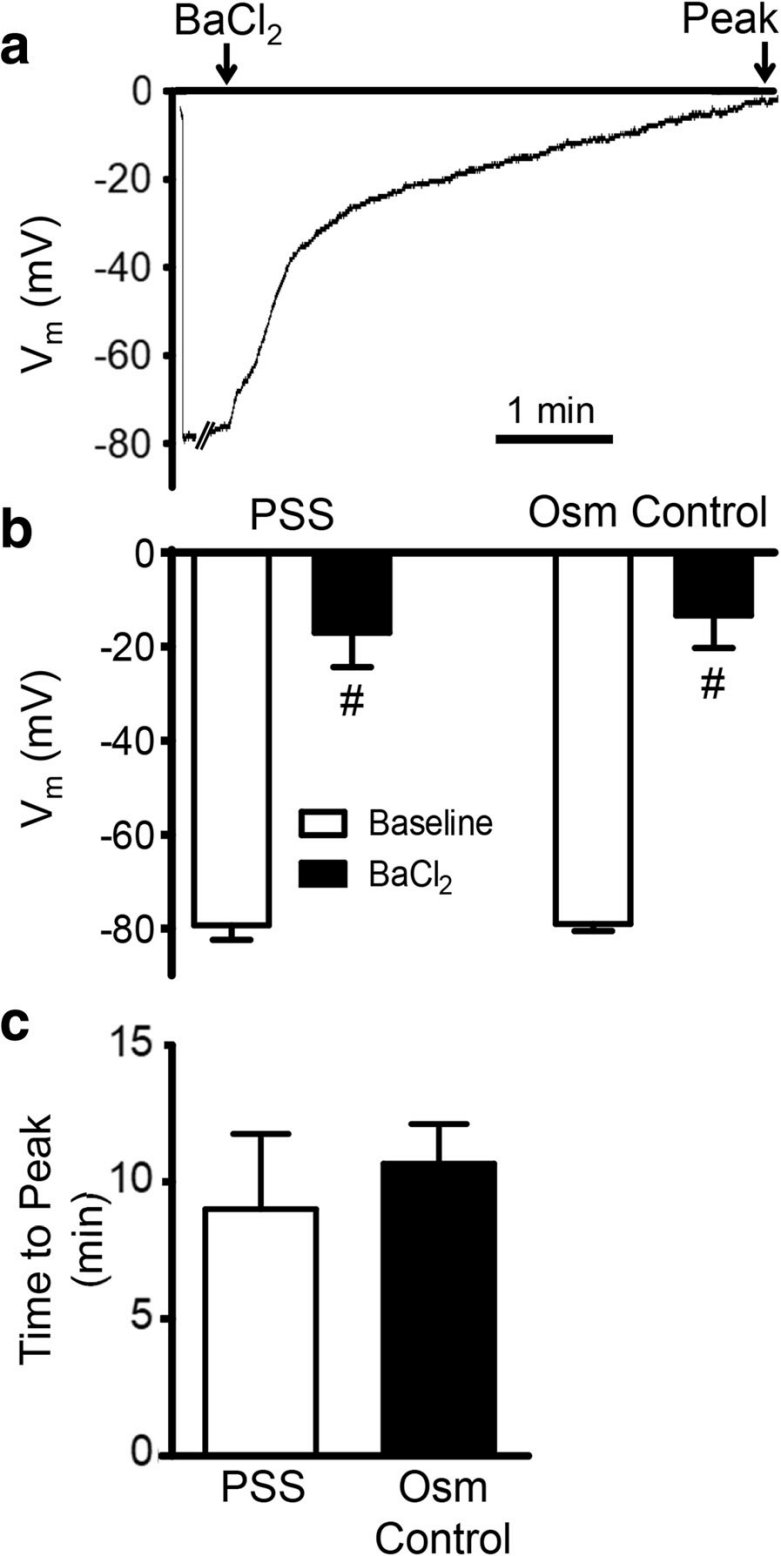
BaCl_2_ depolarizes skeletal muscle myofibers. **a** Representative continuous recording of V_m_ illustrates depolarization of mouse EDL myofiber upon exposure to 1.2% BaCl_2_. **b** Summary data for V_m_ are at resting baseline, at peak depolarization during 1.2% BaCl_2_ added to standard PSS and to PSS in which BaCl_2_ replaced NaCl for osmotic (Osm) control. **c** Summary data for time to peak depolarization during 1.2% BaCl_2_ added to standard PSS, and to PSS in which BaCl_2_ replaced NaCl for Osm control. Values are means ± SEM (*n* = 3–6 myofibers, each from one EDL muscle per mouse). ^#^*P* ≤ 0.05 vs. baseline

Resting V_m_ of EDL myofibers was ~ − 80 mV, consistent with previous reports [27–29]. The myofiber sarcolemma contains multiple K^+^ channels, including K_V_, K_IR_, K_Ca_, and K_ATP_ [30]. Consistent with BaCl_2_ acting as a broad spectrum K^+^ channel inhibitor [12], the addition of 1.2% BaCl_2_ to standard PSS irrigating the muscle depolarized myofibers from − 79 ± 3 mV at rest to − 17 ± 7 mV (Fig. 1; *P* = 0.001). A rapid phase of depolarization occurred within the first 1–2 min followed by a slower phase (Fig. 1*a*). In some cells, V_m_ reached 0 mV indicating cell death. A similar depolarization was recorded when BaCl_2_ was substituted isotonically for NaCl (osmotic control, Fig. 1*b*; *P* = 0.001), illustrating that the effects of BaCl_2_ were not due to osmotic changes from its addition to PSS. There were no differences in ΔV_m_ (vehicle 62 ± 5 mV, osmotic control 66 ± 8 mV; *P* = 0.72), or the time course (Fig. 1*c*; *P* = 0.68) between respective solutions containing 1.2% BaCl_2_. In the absence of BaCl_2_, V_m_ remained stable (~ − 80 mV) for at least 30 min (*n* = 3).

### BaCl_2_ increases [Ca^2+^]_i_ and muscle force

**Fig. 2:**
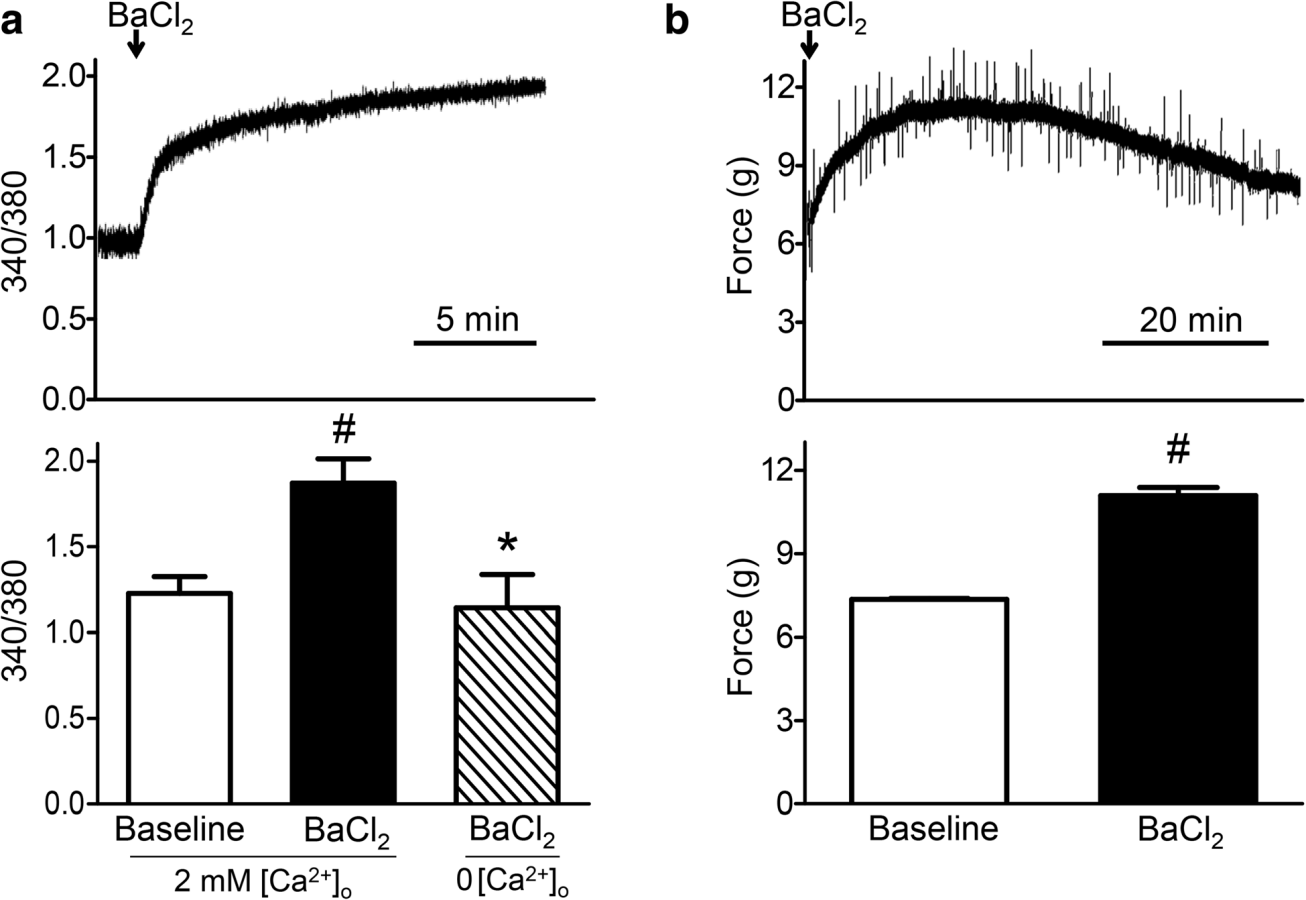
BaCl_2_ increases [Ca^2+^]_i_ and muscle force. **a** Top: representative continuous recording of F_340_/F_380_ illustrates intracellular Ca^2+^ accumulation. Bottom: summary data for F_340_/F_380_ at rest (baseline) and during peak response to 1.2% BaCl_2_ in PSS (*n* = 5) and 1.2% BaCl_2_ in Ca^2+^-free PSS (0 [Ca^2+^]_o_) (*n* = 3). **b** Top: representative continuous recording of force developed by EDL in situ at optimum resting length (*L*_o_) in response to irrigation with 1.2% BaCl_2_ for 1 h. Bottom: summary data for resting and peak force in response to 1.2% BaCl_2_; values are means ± SEM (*n* = 4 muscles). ^#^*P* ≤ 0.05 vs. baseline, *P ≤ 0.05 vs. 1.2% BaCl_2_ in standard PSS with 2 mM extracellular calcium concentration ([Ca^2+^]_o_)

A primary consequence of myofiber depolarization in healthy muscle is internal release of Ca^2+^ from the sarcoplasmic reticulum (SR) via coupling to L-type Ca^2+^ channels (i.e., dihydropyridine receptors), which act as voltage sensors in the sarcolemma [31]. The addition of 1.2% BaCl_2_ to standard PSS evoked a robust increase in myofiber [Ca^2+^]_i_ (Fig. 2*a*; *P* < 0.001). Isotonic BaCl_2_ solution resulted in a similar increase in [Ca^2+^]_i_ (F_340_/F_380_ increased from 1.18 ± 0.02 (baseline) to 1.58 ± 0.06 (BaCl_2_); *n* = 3). In contrast, adding 1.2% BaCl_2_ to Ca^2+^-free PSS had no significant effect on [Ca^2+^]_i_ (Fig. 2*a*). In the absence of BaCl_2_, Fura 2 fluorescence remained stable at the resting baseline for at least 30 min (*n* = 3).

Irrigating the EDL in situ with 1.2% BaCl_2_ in standard PSS increased resting force from 7.4 ± 0.1 to 11.1 ± 0.4 g over ~ 30 min, which then returned to baseline during the 60 min exposure (Fig. 2*b*; *P* = 0.001). Whereas a rise in [Ca^2+^]_i_ activates the contractile proteins [32], sustained elevation of [Ca^2+^]_i_ stimulates mitochondrial production of reactive oxygen species (ROS), which can impair cross-bridge function [33]. Ca^2+^-activated proteolysis disrupts the integrity of contractile proteins [15], which we surmise may have occurred in the present experiments.

### BaCl_2_ activates proteolysis and disrupts membranes

**Fig. 3:**
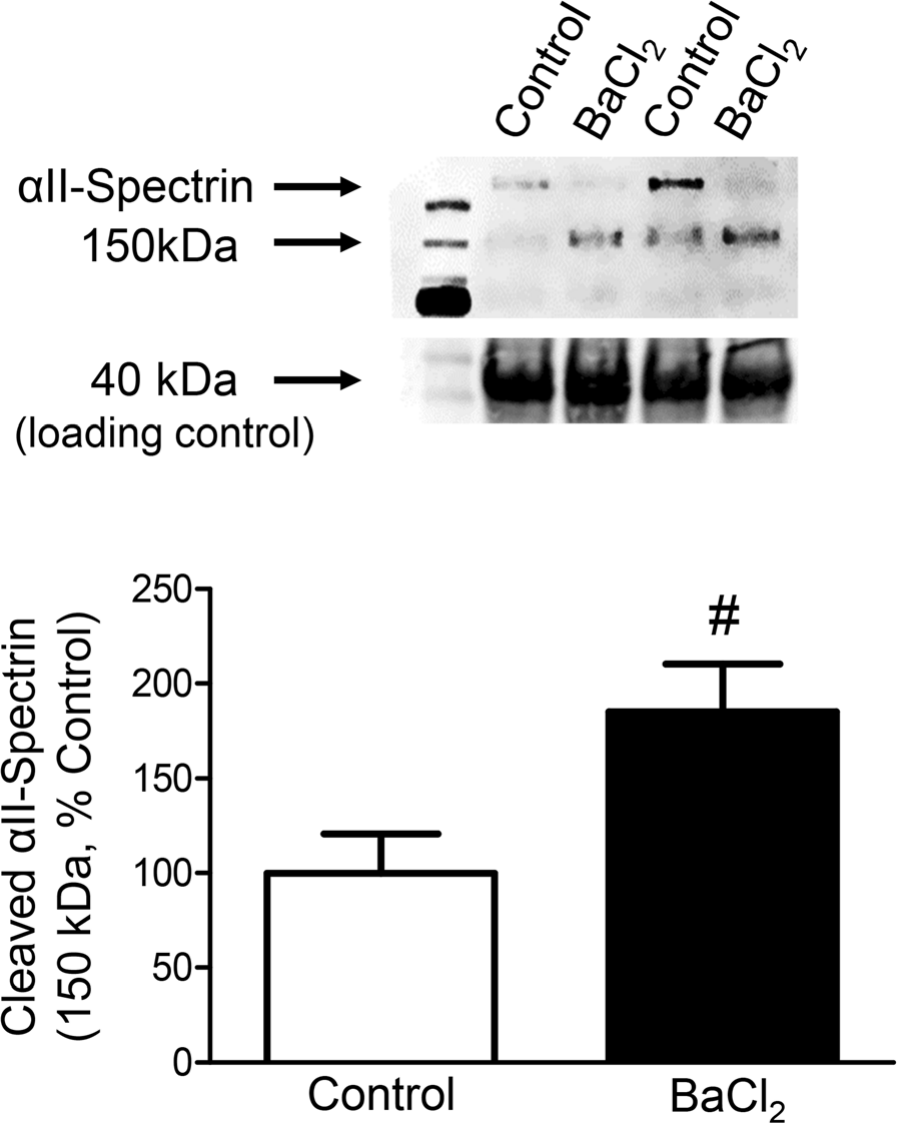
BaCl_2_ increases calpain activity. Representative Western blots (top) and mean densitometric data (bottom) for αII-spectrin from EDL muscles treated with standard PSS (Control) or 1.2% BaCl_2_ in standard PSS for 1 h. The αII-spectrin band at 240 kDa and its cleavage product at 150 kDa were both normalized to total protein reflected by the 40 kDa band, which was not different between samples. Summary data are means ± SEM (*n* = 6 muscles). ^#^*P* ≤ 0.05 vs. control

Elevating [Ca^2+^]_i_ leads to degradation of muscle fibers through proteolysis by Ca^2+^-activated neutral proteases [15, 16]. For example, calpain is activated in two primary steps: (1) the inactive enzyme translocates to the sarcolemma where the N-terminus is cleaved through autolysis releasing active calpain, and (2) two Ca^2+^ ions bind to the protease domain to maintain the active site [34]. Active calpain cleaves skeletal muscle structural proteins including titan, nebulin, and αII-spectrin [35]. In EDL muscles exposed to 1.2% BaCl_2_ in standard PSS for 1 h, αII-spectrin was cleaved from 240 to a 150 kDa product (Fig. 3; *P* = 0.02), which was accompanied by an increase in the ratio of cleaved: total αII-spectrin (control = 2.8 ± 1.25; BaCl_2_ = 17.9 ± 8.9 (*P* = 0.12, *n* = 6)).

**Fig. 4:**
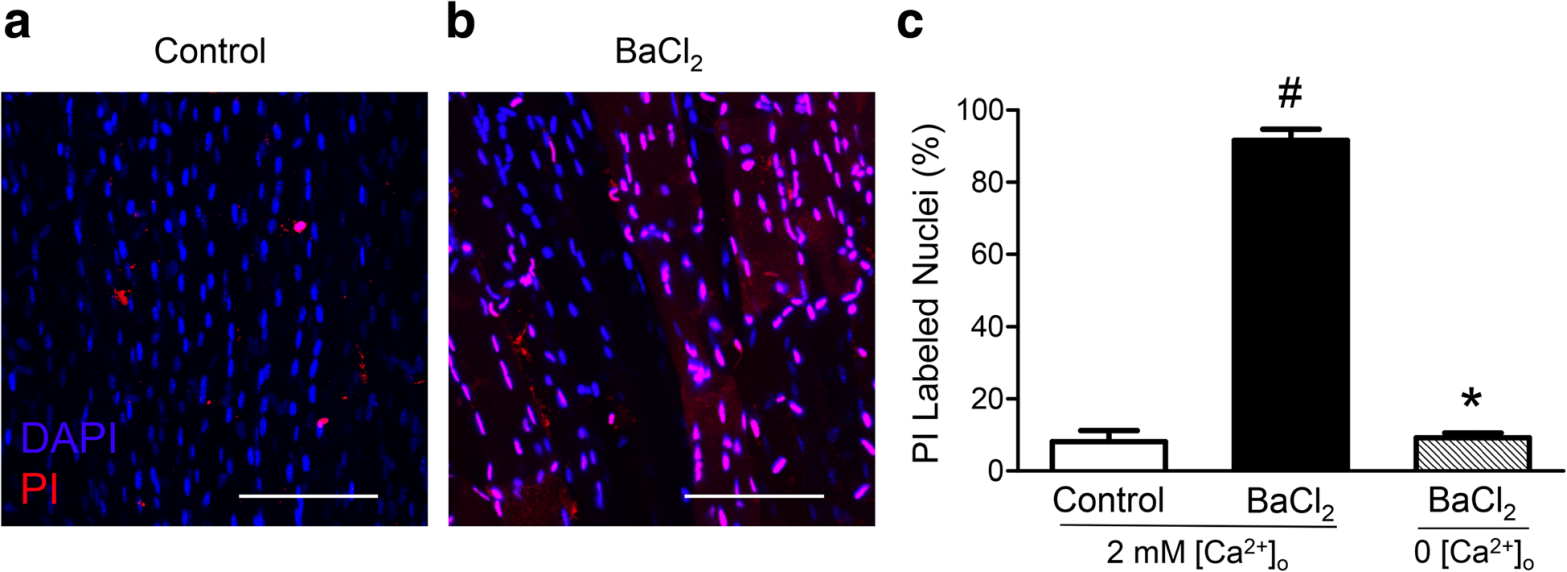
BaCl_2_ disrupts myofiber plasma membranes. **a**, **b** Representative images of nuclear staining in EDL muscles after 1 h in standard PSS (control) and in 1.2% BaCl_2_ added to standard PSS. Propidium iodide (PI, red) identifies nuclei of cells with disrupted plasma membranes and Hoechst 33342 (blue) is membrane permeant and identifies all cell nuclei. Removal of extracellular Ca^2+^ (0 [Ca^2+^]_o_)ameliorated myonuclear staining with PI as in **a**. **c** Summary data for percentage of PI-labeled nuclei, calculated as (# red nuclei/# blue nuclei) × 100. Each region of interest contained ~ 150 myonuclei. Summary data are means ± SEM for *n* = 4–5 muscles per group. ^#^*P* ≤ 0.05 vs. control, *P ≤ 0.05 vs. 1.2% BaCl_2_ in standard PSS with 2 mM [Ca^2+^]_o_. Scale bars, 100 μm

Myonuclei in EDL muscles treated with PSS exhibited minimal PI staining after 1 h (8%) while nearly all myonuclei (92%) were stained following 1 h of exposure to 1.2% BaCl_2_, thus indicating gross disruption of sarcolemma throughout the muscle (Fig. 4; *P* < 0.001). Removal of Ca^2+^ from the irrigation solution prevented the incorporation of PI, demonstrating the importance of Ca^2+^ influx from the extracellular fluid in BaCl_2_-induced membrane disruption (Fig. 4*c*; *P* < 0.0001). Ca^2+^-activated calpain can stimulate several proapoptotic pathways. While calpain-activated caspase 12 promotes apoptosis through the “executioner” caspase 3 [36, 37], it can also cleave BH3-only protein (Bid) and apoptosis-inducing factor (AIF) to truncated Bid and truncated AIF, thereby causing mitochondrial membrane permeability and ensuing apoptosis [38].

### BaCl_2_ induces injury of motor axons and microvessels

**Fig. 5:**
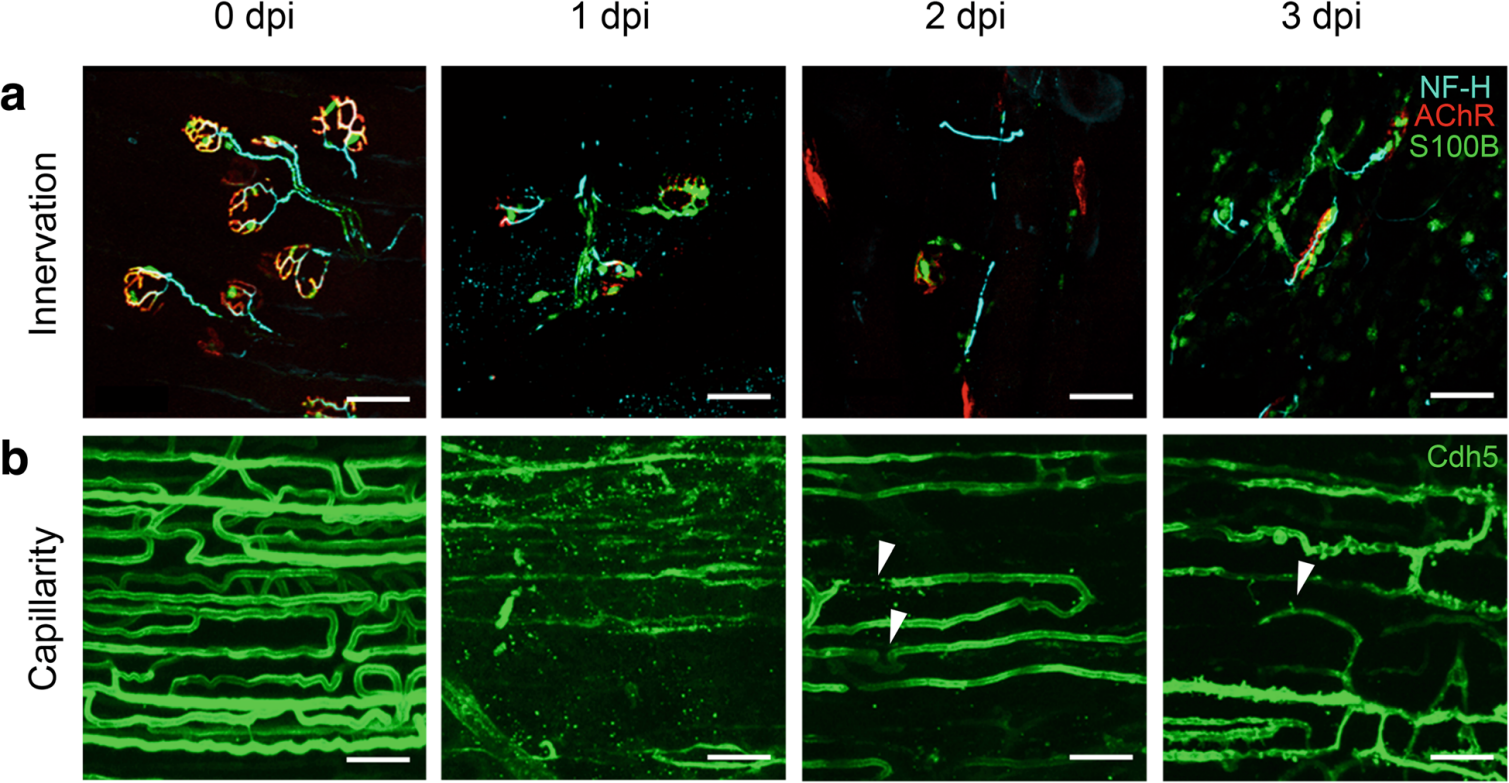
Motor innervation and capillaries are disrupted by local BaCl_2_ injection. **a** Neuromuscular junctions in TA muscle. Schwann cells (green) are closely associated with axonal neurofilament-heavy (cyan; indicates motor axons) and overlay postsynaptic nicotinic receptors (red) at 0 dpi (uninjured control). Following injection of 1.2% BaCl_2_, NMJ components are dissociating at 1 dpi and fragmented at 2 and 3 dpi. **b** Capillaries in GM (green endothelial cells) are densely organized and align along myofibers in uninjured muscle (0 dpi). Following injection of 1.2% BaCl_2_, disrupted and fragmented capillaries are observed at 1–2 dpi (arrowheads) while evidence of capillary neoformation is apparent at 3 dpi (arrowhead). Scale bars, 50 μm. Color coding in 3 dpi panels applies to earlier timepoints for innervation and capillarity. NF-H, neurofilament heavy; AChR, nicotinic actetylcholine receptors; S100B, Schwann cells expressing GFP; Cdh5, endothelial cells expressing GFP

In contrast to the integrity of pre- and postsynaptic elements characteristic of healthy NMJs, neurofilament-heavy staining at 1 dpi appeared fragmented, suggesting axonal disruption. Clusters of acetylcholine receptors also became dispersed along the laminar surface and Schwann cells began to migrate away from the NMJ, which progressed through 2 dpi (Fig. 5*a*). By 3 dpi, Schwann cells appear to associate with axonal fragments and AChR clusters. These data demonstrate that motor axons in the vicinity of BaCl_2_ injection undergo degeneration within 24 h that extends over 3 days, consistent with the time course of Schwann cell migration following axotomy [39].

Uninjured muscle exhibits an orderly network of capillaries (Fig. 5*b*). Following BaCl_2_ injury, capillaries were fragmented at 1 dpi. By 3 dpi, anastomoses (interconnecting loops) began to appear between capillary sprouts. While these observations add new insight to the extent of tissue injury induced by BaCl_2_, our findings are consistent with structural damage of microvessels induced by BaCl_2_ at 2 dpi [3] and our recent report that capillary perfusion was disrupted at 1 dpi [8].

## Discussion

Skeletal muscle comprises ~ 40% of body mass and has the remarkable ability to regenerate following injury due to resident satellite cells. Skeletal muscle injuries occur in multiple ways including disease, physical trauma, temperature extremes, eccentric contractions, and exposure to myotoxic agents [3–5]. Whereas myofibers follow a similar pattern of regeneration irrespective of the mechanism of injury [1, 40], the kinetics and involvement of satellite cells can vary with the nature of insult [3]. For example, freeze injury results in a dead zone of tissue that viable cells must penetrate, whereas local exposure to BaCl_2_ induces coordinated necrosis of myofibers with infiltration of inflammatory cells followed by sequential regeneration of myofibers [1, 3].

Unlike freeze damage, BaCl_2_-induced injury preserves satellite cells, which allows detailed examination of their gene expression, cell signaling, and regeneration kinetics in vivo. Remarkably, how BaCl_2_ kills myofibers has remained undefined. In accord with the ability of Ba^2+^ to block K^+^ channels [11, 12], we reasoned that it would depolarize myofibers, as the sarcolemma contains K_V_, K_IR_, K_Ca_, and K_ATP_ channels [30]. The progression of depolarization we observed in EDL may reflect reliance on Cl^−^ conductance for resting V_m_ in skeletal muscle, which can buffer the abrupt effect of changing the conductance of other ions [41]. Following BaCl_2_-induced depolarization, the present data show that increasing [Ca^2+^]_i_ leads to proteolysis, membrane disruption, and myofiber death. Moreover, preventing the rise of [Ca^2+^]_i_ by removing extracellular Ca^2+^ preserves membrane integrity as demonstrated by the paucity of myonuclei stained with PI under this condition (Fig. 4c). Myotoxicity through Ca^2+^-mediated proteolysis and membrane disruption subsequent to Ba^2+^ exposure is consistent with the action of biological agents known to disrupt myofibers such as bee, wasp, and snake venoms [42–44].

BaCl_2_ has been used to study the pathophysiology of hypokalemia, a clinical condition which depolarizes muscle fibers through reduced K^+^ efflux [10, 45]. In hypokalemia, the SR is integral to myofiber disruption [10, 46, 47], with Ca^2+^ release from internal stores being a primary source of the elevated [Ca^2+^]_i_ that contributes to muscle injury. While the relative contribution of Ca^2+^ release from internal stores vs. influx through L-type channels during BaCl_2_ injury remains to be determined, the SR is the principal source of elevating [Ca^2+^]_i_ during muscle contractions [48]. Consistent with this effect, we observed transient contraction of the EDL upon exposure to BaCl_2_ that peaked with the rise in [Ca^2+^]_i_ over 20–30 min (Fig. 2). The recovery to resting (passive) tension during the ensuing 30 min may reflect disruption of the contractile machinery. This interpretation is consistent with the degradation of αII-spectrin we observed within 60 min of BaCl_2_ exposure (Fig. 3). Once in the cytoplasm, Ba^2+^ can enter mitochondria [49] and generate superoxide by increasing electron flow from Ca^2+^-sensitive citric acid cycle dehydrogenases and thereby dissipate mitochondrial membrane potential [50]. The ensuing disruption of mitochondria releases cytochrome C into the cytosol to initiate intrinsic apoptosis, culminating in the activation of caspase 3 and cell death [36].

### Nerve and microvessel injury

Motor nerves and microvessels control and supply myofibers of intact skeletal muscle by initiating contraction and delivering nutrients in response to metabolic demand [51]. Given their intimate physical proximity and shared signaling events, we hypothesized that muscle injury induced by BaCl_2_ would disrupt motor axons and capillaries. Similar to the time course of myofiber disruption [3], the present data illustrate that motor nerves and microvessels appear fragmented within 24 h following local injection of BaCl_2_ (Fig. 5).

It is unclear whether nerves and capillaries undergo damage directly from BaCl_2_ or indirectly as a secondary effect of myofiber disruption. Mechanical changes within the injured myofiber can lead to degeneration of the NMJ. For example, with local injury, myofilaments contract on both sides of the injured site, leaving an empty tube with partially retracted nerve terminals juxtaposed to the site [52]. While mechanisms of axon retraction remain to be defined, the change in cell shape suggests that it is a consequence of cytoskeletal remodeling in response to a retraction program or loss of the ability to maintain the cytoskeleton [53]. Because it is a cytoskeletal protein integral to the structure of cell membranes, degradation of αII-spectrin is disruptive to the sarcolemma and contributes to fragmentation of motor nerve synapses with dissolution of AChR clusters (Fig. 5).

Disruption of capillaries occurs in multiple models of muscle injury [3]. The present data are the first to illustrate that these events coincide with the loss of neuromuscular integrity. Thus, key elements of myofiber control and supply are similarly affected, with loss of structural integrity occurring during the initial 24 h (1 dpi) and initial stages of recovery apparent at 3 dpi. In addition to myofibers, vessels and nerves may undergo calpain-dependent degradation [54, 55]. Thus, while skeletal muscle consists primarily of myofibers, the increase in calpain-specific αII-spectrin degradation measured in our homogenates (Fig. 3) may be derived from multiple cell types. As we have observed directly with intravital microscopy in the GM, the inflammatory response to BaCl_2_ begins within 1–2 h of exposure (Fernando and Segal, unpublished observations from [8]). Infiltration of the tissue with neutrophils, monocytes, and pro-inflammatory macrophages ensues over the next 2–3 days, thereby disrupting all tissue components indiscriminately [3, 56] through activation of additional proteolytic pathways and oxidative modification of proteins to accelerate proteolysis [36].

## Conclusion

Skeletal muscle injury induced by BaCl_2_ is widely used as a method for studying myofiber damage and regeneration [3, 6–9]. Because the mechanism of BaCl_2_-induced injury was unknown, the goal of the present study was to define the nature of myofiber damage and ascertain whether associated tissue elements were similarly affected. Using complementary ex vivo preparations of skeletal muscle, we demonstrate that acute exposure to BaCl_2_ causes myofiber damage via Ca^2+^-dependent proteolysis secondary to membrane depolarization. Further, motor axons and microvessels appear to undergo damage with a similar time course to the disruption of myofibers. These data provide a foundation for investigating how major tissue components responsible for skeletal muscle structure and function (i.e., myofibers, motor nerves, and microvessels) respond to and interact during muscle injury and regeneration.

## Supplementary information

**Additional file 1.** Satellite cells are spared from BaCl_2_-induced death in vitro. Treatment with BaCl_2_ in vivo is used to induce myofiber damage leading to satellite cell activation and muscle regeneration. To formally demonstrate the differential effects of BaCl_2_ on myofibers and their associated satellite cells, we isolated single muscle fibers from the EDL muscle and exposed them to either saline or 1.2% BaCl_2_ in the presence of propidium iodide (PI) to label nuclei with disrupted laminae (dead or dying). Fibers were fixed in 4% paraformaldehyde then stained for expression of the satellite cell marker CD34 (rat monoclonal RAM-34, eBioscience at 1:200) (arrows). Myofibers fixed at 0 min (*A*) or after 50 min in Ca^2+^, Mg^2+^-free PBS (*B*) retain their morphology and integrity and do not incorporate PI in either satellite cell nuclei or myonuclei. *In contrast, m*yofibers exposed to 1.2% BaCl_2_ for 50 min (*C*) have hypercontracted, lost their structural integrity, and possess PI-labeled nuclei. However, satellite cells associated with these fibers have not incorporated PI. *D*, Addition of 1.2% BaCl_2_ to isolated single fibers leads to elevated [Ca^2+^]_i_ as observed in whole-muscle preparations (Fig. 2). The severity and kinetics of BaCl_2_-induced myotoxicity for single fibers appear to be less than observed in our experiments using whole muscles. We hypothesize that the absence of fixed attachments at the ends of single myofibers may reduce the damaging membrane stress following Ca^2+^-induced hypercontraction. Scale bars = 10 μm.

## Data Availability

The datasets used and/or analyzed during the current study are available from the corresponding author on reasonable request. Materials used in this study are commercially available.

## Abbreviations

[Ca^2+^]_i_: Intracellular calcium concentration
[Ca^2+^]_o_: Extracellular calcium concentration
AChR: Acetylcholine receptors
AIF: Apoptosis-inducing factor
Bid: BH3-only protein
dpi: Days post injury
EDL: Extensor digitorum longus
EGTA: Ethylene glycol-bis (β-aminoethyl ether)-N,N,N′,N′-tetraacetic acid
GFP: Green fluorescent protein
GM: Gluteus maximus
IgG: Immunoglobulin G
NMJ: Neuromuscular junction
PBS: Phosphate-buffered saline
PSS: Physiological salt solution
ROI: Region of interest
TA: Tibialis anterior
V_m_: Membrane potential
