# Supplementary figures and images for: Barium chloride injures myofibers through calcium-induced proteolysis with fragmentation of motor nerves and microvessels
Source: Skelet Muscle. 2019 Nov 6;9:27. doi: 10.1186/s13395-019-0213-2 (PMC6833148; doi:10.1186/s13395-019-0213-2)

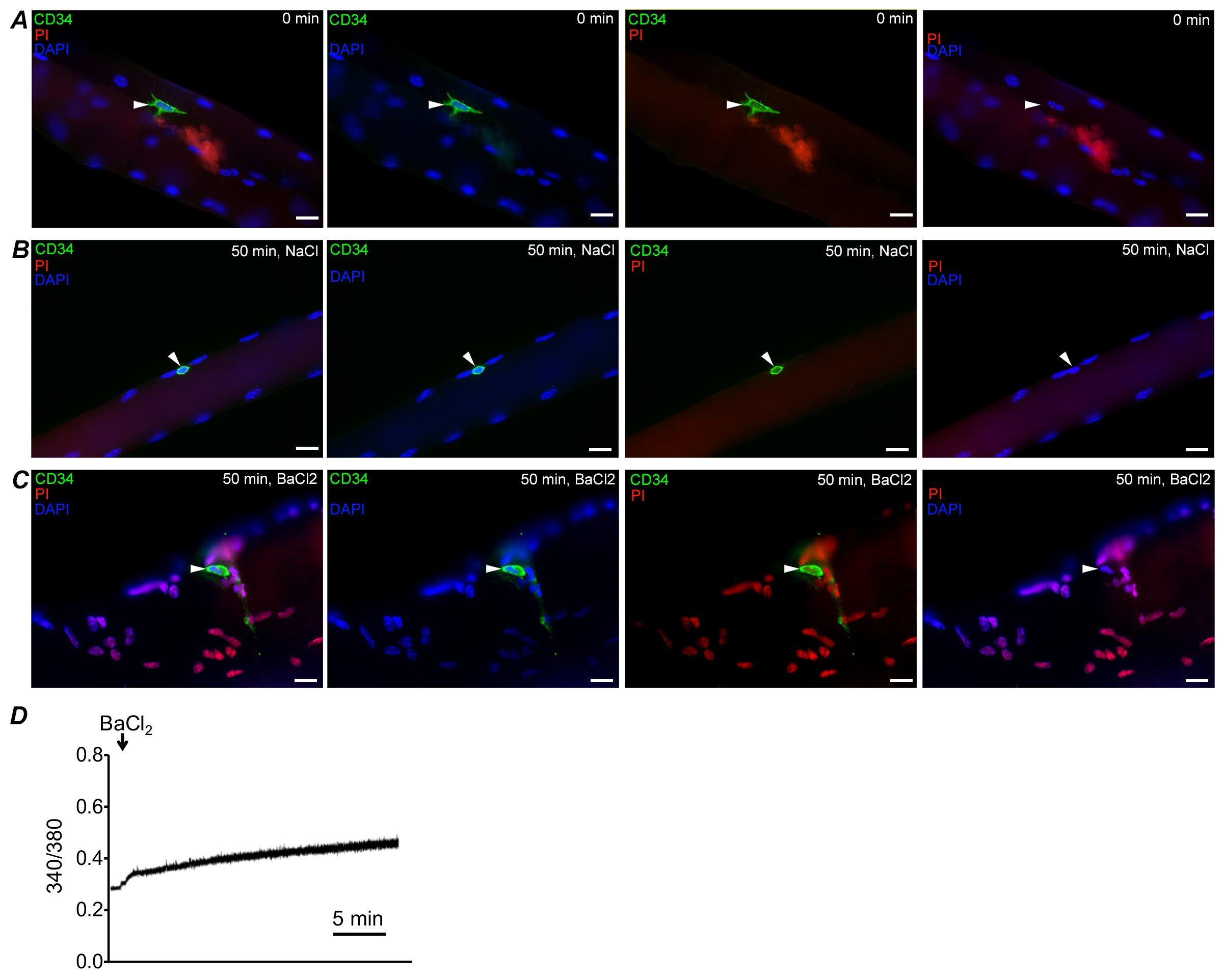

Supplement: Supplementary file 1 — Additional file 1. Satellite cells are spared from BaCl2-induced death in vitro. Treatment with BaCl2 in vivo is used to induce myofiber damage leading to satellite cell activation and muscle regeneration. To formally demonstrate the differential effects of BaCl2 on myofibers and their associated satellite cells, we isolated single muscle fibers from the EDL muscle and exposed them to either saline or 1.2% BaCl2 in the presence of propidium iodide (PI) to label nuclei with disrupted laminae (dead or dying). Fibers were fixed in 4% paraformaldehyde then stained for expression of the satellite cell marker CD34 (rat monoclonal RAM-34, eBioscience at 1:200) (arrows). Myofibers fixed at 0 min (A) or after 50 min in Ca2+, Mg2+-free PBS (B) retain their morphology and integrity and do not incorporate PI in either satellite cell nuclei or myonuclei. In contrast, myofibers exposed to 1.2% BaCl2 for 50 min (C) have hypercontracted, lost their structural integrity, and possess PI-labeled nuclei. However, satellite cells associated with these fibers have not incorporated PI. D, Addition of 1.2% BaCl2 to isolated single fibers leads to elevated [Ca2+]i as observed in whole-muscle preparations (Fig. 2). The severity and kinetics of BaCl2-induced myotoxicity for single fibers appear to be less than observed in our experiments using whole muscles. We hypothesize that the absence of fixed attachments at the ends of single myofibers may reduce the damaging membrane stress following Ca2+-induced hypercontraction. Scale bars = 10 μm. [file 13395_2019_213_MOESM1_ESM.tif]
